# Supplementary figures and images for: Concentration-dependent polymorphism of insulin amyloid fibrils
Source: PeerJ. 2019 Dec 10;7:e8208. doi: 10.7717/peerj.8208 (PMC6910113; doi:10.7717/peerj.8208)

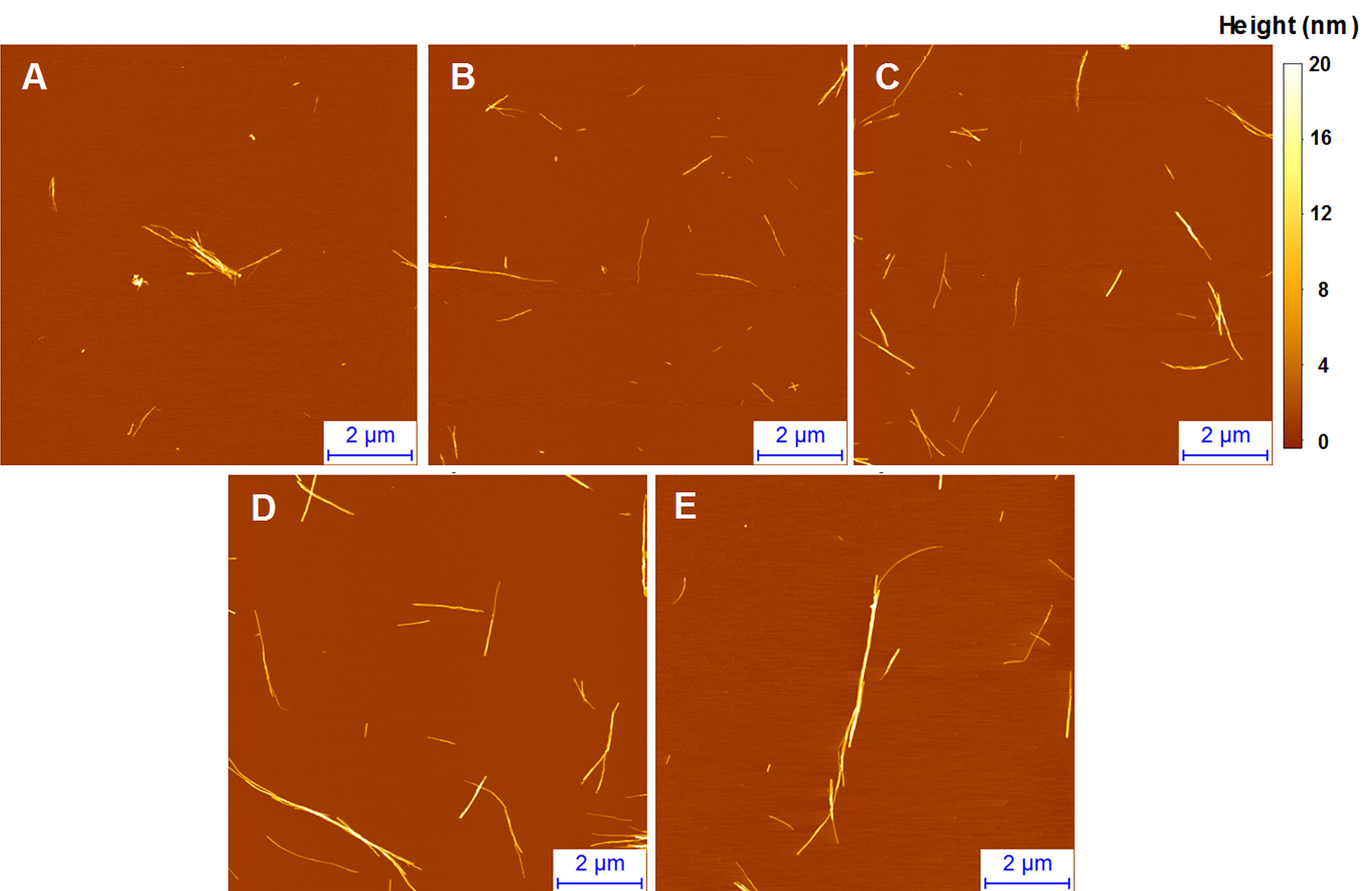

Supplement: Figure S1 — Formed at 0.2 mM (A), 0.4 mM (B), 0.6 mM (C), 0.8 mM (D) and 1.0 mM (E) concentrations. [file peerj-07-8208-s001.png]

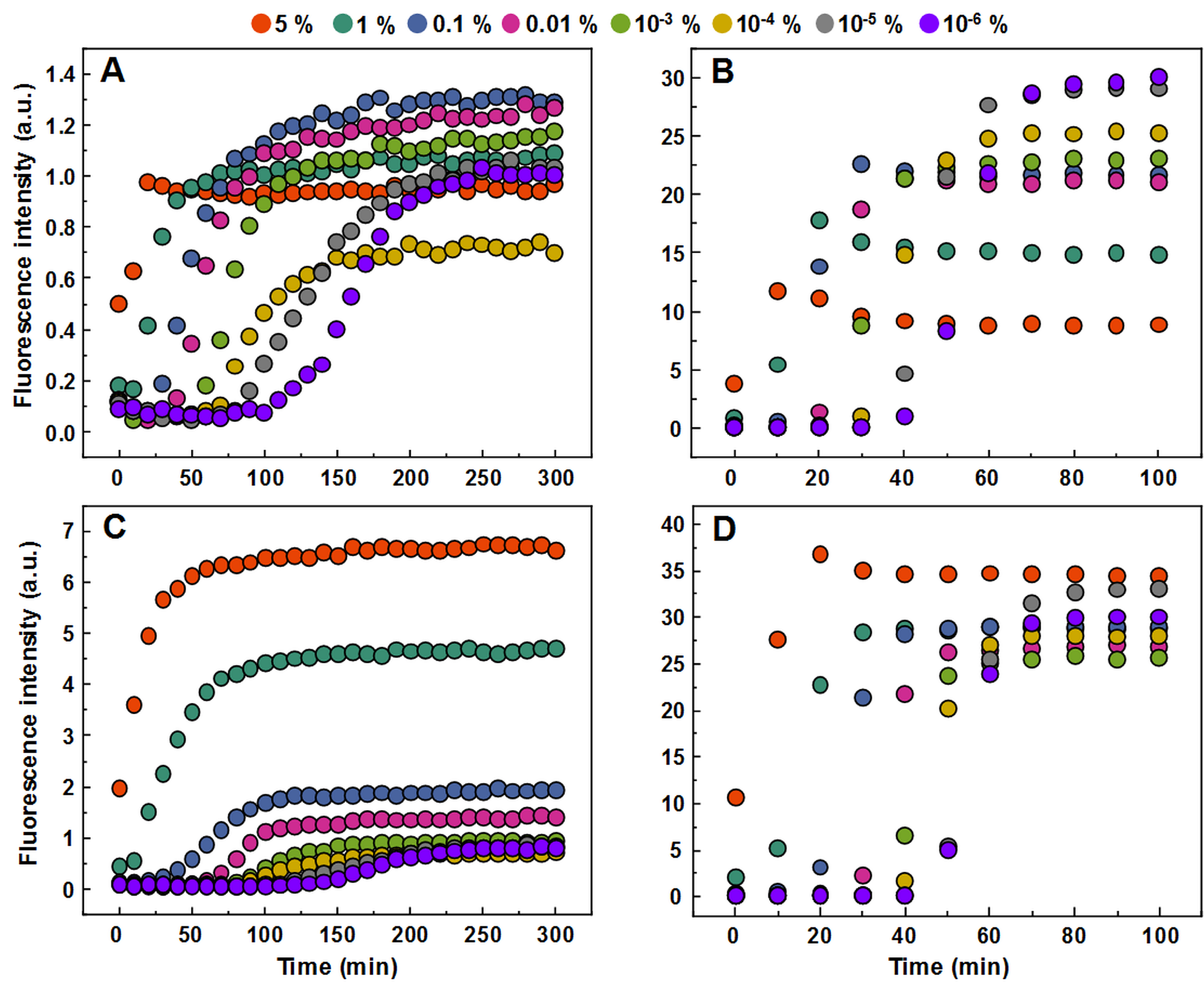

Supplement: Figure S1 — Aggregation kinetics of insulin where the LCF are added to 0.2 mM insulin solutions (A), LCF to 1.0 mM (B), HCF to 0.2 mM (C) and HCF to 1.0 mM (D). [file peerj-07-8208-s002.png]

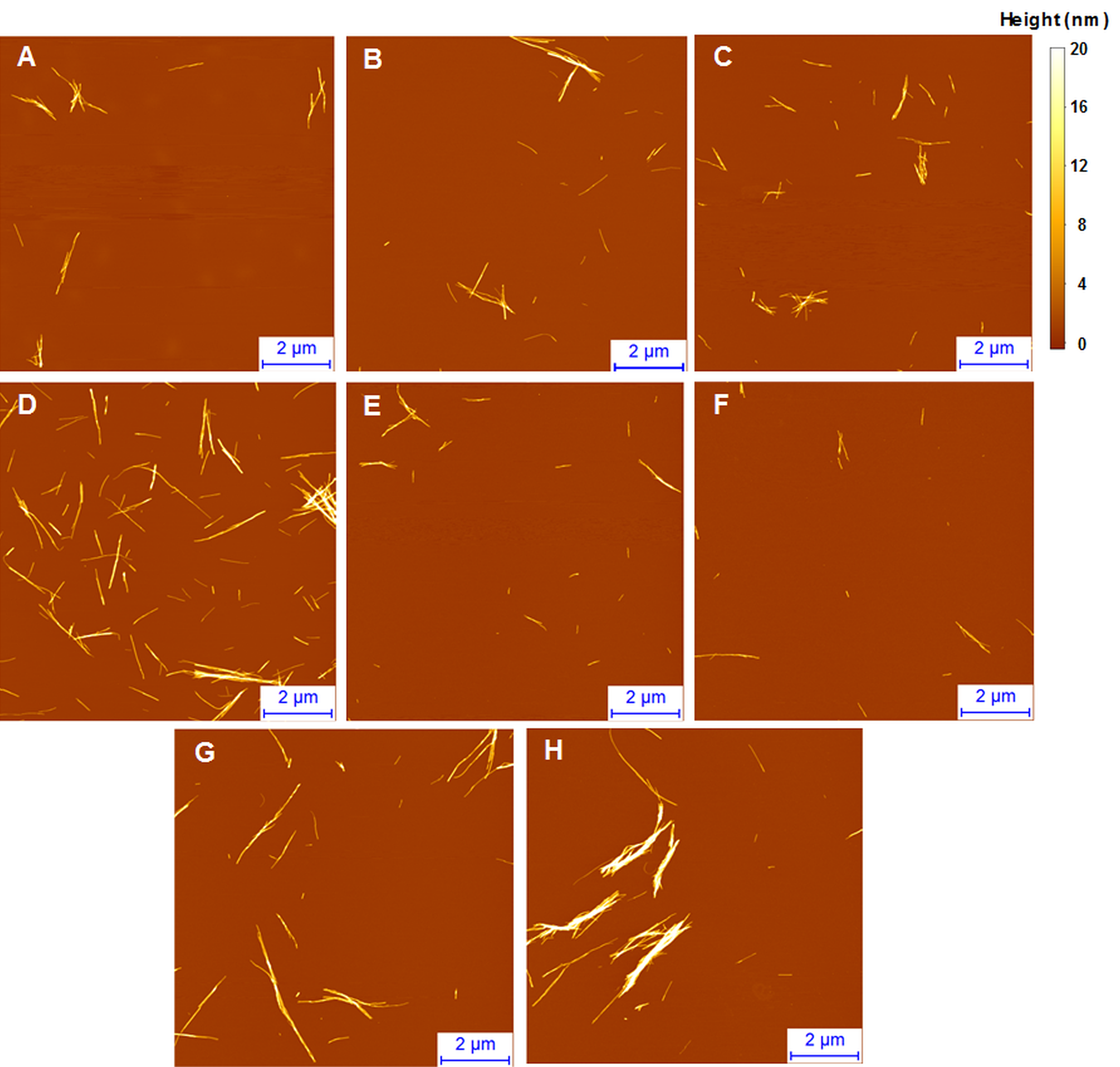

Supplement: Figure S3 — Insulin fibrils resulting from seeding 0.2 mM insulin with LCF (A, E), 0.2 mM with HCF (B, F), 1.0 mM with LCF (C, G), 1.0 mM with HCF ( D, H) using 5% or 10−4 % of preformed fibrils respectively. [file peerj-07-8208-s003.png]
